# The Role of Gender in Juvenile Idiopathic Arthritis-Associated Uveitis

**DOI:** 10.1155/2014/461078

**Published:** 2014-02-20

**Authors:** Ahmadreza Moradi, Rowayda M. Amin, Jennifer E. Thorne

**Affiliations:** ^1^The Division of Ocular Immunology, Department of Ophthalmology, The Wilmer Eye Institute, Johns Hopkins University School of Medicine, 600 North Wolfe Street, Woods Building, Room 476, Baltimore, MD, USA; ^2^Department of Epidemiology, Johns Hopkins University Bloomberg School of Public Health, Baltimore, MD, USA

## Abstract

Uveitis is a common complication of juvenile idiopathic arthritis (JIA) affecting up to 30% of patients with JIA. Although the typical bilateral chronic anterior uveitis associated with the persistent and extended oligoarticular and polyarticular, rheumatoid factor negative variants of JIA occurs predominantly in girls, boys may be more commonly affected in the HLA-B27 positive, enthesitis variant of JIA. While female gender has been associated with the development of the chronic anterior uveitis in children with JIA, the clinical course of JIA-associated uveitis may be worse in boys than in girls. The purpose of this paper is to review the available published literature to determine the role of gender in the clinical presentation and outcomes of patients with JIA-associated uveitis.

## 1. Introduction

Juvenile idiopathic arthritis (JIA) represents a heterogeneous group of chronic arthritides that affect children aged 16 years and under [1–3]. Uveitis is a common complication of JIA, occurring in upwards of 30% of patients who are positive for antinuclear antibody (ANA) [3]. The most typical type of uveitis is a chronic bilateral anterior uveitis with insidious onset and persistent duration. It is this type of uveitis that is thought to confer the greatest risk for structural ocular complications and visual acuity loss among children suffering from JIA-associated uveitis because of its insidious onset, the lack of a red eye, and the onset in preverbal children. However, other types of JIA-associated uveitis may occur, such as the recurrent acute anterior uveitis associated with enthesitis and a positive HLA-B27. These cases typically present as an acutely red, painful and photophobia eye and occur more commonly in boys than in girls. Alternatively, the typical chronic anterior uveitis associated with JIA occurs predominantly in girls. Furthermore, not only does the proportion of girls versus boys vary according to the type of arthritis and the type of uveitis, it is possible that the clinical outcomes in girls and boys may differ within the same subsets of JIA uveitis. The purpose of this paper therefore is to summarize the available literature investigating the role of gender in the clinical and treatment outcomes in JIA-associated uveitis.

## 2. Prevalence and Incidence of JIA and JIA-Associated Uveitis

Multiple cohort and population-based studies have reported the prevalence and/or incidence of juvenile idiopathic arthritis in populations aged 16 and under and are summarized in Table 1. In general, the prevalence of JIA ranges from 16 to 140 per 100,000 population [4–9, 11]. The incidence of JIA ranges from 3 to 23 per 100,000 person-years (PY) [4–8, 11]. The prevalence of JIA-associated uveitis is estimated as high as 30 per 100,000 people in North America and Europe, with incidence rates ranging from 4.3 to 6 per 100,000 PY [11–13]. It has been reported that up to 30% of patients thought to be at high risk for developing uveitis (ANA positivity, oligoarticular disease, female gender) will in fact go on to develop chronic anterior uveitis associated with JIA, with approximately 90% of patients being diagnosed within the first five years after the diagnosis of the joint disease [1–3, 12]. Interestingly one study from Italy suggested that the presence of ANA is the strongest risk factor for developing uveitis, with 30% of ANA positive children with JIA developing uveitis regardless of arthritis type and gender [3]; however, other studies [2, 5, 11, 14] have not duplicated these findings. Registry data from Canada [14] and Germany [15] have suggested that the development of uveitis occurs in the smaller proportion of patients, perhaps on the order of 10–15%. This could be due to the use of disease modifying antirheumatic drugs (DMARDs) to treat the joint disease in the last decade lowering the proportion of patients that ultimately develop uveitis or due to the inclusion of children with milder forms of JIA who perhaps are less likely to develop extra-articular complications of JIA such as uveitis. The epidemiologic data typically have not investigated differences between gender in terms of the prevalence and incidence of uveitis; however, certain types of JIA such as enthesitis-related JIA where patients often have the HLA-B27 haplotype typically affect boys more commonly than girls. The estimated ratio of boys to girls in enthesitis-related JIA is 1.5 : 1 [16]. In contrast, girls typically out number boys in the other JIA subtypes in which uveitis is likely to occur, such as persistent and extended oligoarticular disease and polyarticular, rheumatoid factor negative arthritis [14, 15]. Some of the well-documented risk factors for developing uveitis in children with JIA—specifically younger age, female gender, and positive ANA—form the basis for the uveitis screening recommendations for children with JIA [17]. However, data from a Canadian registry of 1047 patients with JIA suggest that the risk of developing uveitis is age-dependent for girls but not so for boys. Although overall the presence of ANA was the strongest single risk factor for developing uveitis in this study, the proportion of boys with uveitis who were ANA positive was statistically significantly less than the proportion of girls with uveitis who were ANA positive (50% versus 73%, boys versus girls, *P* < 0.001). Taken together, the Canadian registry data suggest an interplay between ANA, age, and gender in role of uveitis screening although validity data are not available for these screening recommendations [18].

## 3. Clinical Presentation

Although the majority of data regarding JIA-related uveitis focuses on patients with chronic bilateral anterior uveitis, HLA-B27-associated recurrent unilateral anterior uveitis may occur associated with the enthesitis subtype of JIA and, as mentioned above, is more common in boys than in girls. Children with this subtype of uveitis typically follow a clinical presentation and course similar to that observed in adult patients with HLA-B27-associated uveitis independent of arthritis. Although these patients typically have recurrent and unilateral uveitis, some patients may evolve into chronic uveitis, and others may develop a bilateral albeit asynchronous uveitis. Interestingly, data from HLA-B27 uveitis in adults suggest that atypical courses of the uveitis including evolution to chronic disease and the development of bilateral uveitis occur more frequently in women than in men; however, the data among children are limited [16].

In terms of the clinical presentation of the chronic anterior uveitis associated with JIA, the majority of children that present to tertiary care clinics (from where most of the published data are reported) already have at least one ocular complication at the time of presentation. The literature from tertiary care centers reports that approximately 50 to 70% of eyes will have at least one ocular complication at presentation, including band keratopathy, cataract, elevated eye pressure, glaucoma, macular edema, optic nerve edema, and epiretinal retinal membrane [19, 20]. In the Johns Hopkins cohort of 75 patients, 67% of patients had at least one ocular complication at the time of presentation. The median time from diagnosis of uveitis to presentation in the Hopkins clinic was approximately six years [20]. Furthermore, approximately one-third of eyes had 20/50 or worse vision at presentation and one-quarter of eyes presented with 20/200 or worse vision. Although the main focus of this paper was not about the role of gender in the presentation of JIA-associated uveitis, the authors investigated the risk factors (including gender) for presenting with poor visual acuity and for presenting with uveitis-related ocular complications [20]. In both univariate and multivariate analyses, gender was not a statistically significant risk factor for any of these outcomes [20]. Because other series have suggested that boys present with more severe uveitis [21–23], we performed subanalyses specific to the role of gender in the Johns Hopkins cohort. Although overall we did not find that boys presented with more severe uveitis than girls, there was a suggestion that in earlier years of the cohort (e.g., patients that presented to the clinic prior to 2000 compared to those presenting in the year 2000 or after), boys tended to have more severe uveitis as evidenced by a higher frequency of posterior synechiae at presentation [20]. However, it is possible that this observation was confounded by the fact that boys experienced a delay in referral to our clinic relative to girls as boys had longer disease durations at the time of presentation during this time period as well. Furthermore, no differences according to gender were reported in a study of 327 patients with JIA-associated uveitis from the Systemic Immunosuppressive Therapy for Eye Disease (SITE) Research Group. This multicenter retrospective study that includes 5 tertiary care centers reported on data collected at the initial visit to the SITE study centers and on subsequent visits; thus, the majority of patients had been diagnosed with JIA-associated uveitis prior to the initial SITE visit and a referral bias likely exists (Table 2) [19].

However, studies both in the UK and The Netherlands have suggested that boys may present with a more severe clinical picture and were more likely to have ocular complications at presentation than were girls [21, 23, 25] (Table 3). These studies were undertaken in combined secondary and tertiary settings so the issue of uveitis duration prior to the presenting examination in these studies is less of a concern and add support to the observation that boys may present with more severe disease independent of disease duration or referral bias—a finding that could not be fully supported by data taken from the strictly referral-based studies described above.

## 4. Incidence of Ocular Complications and Visual Acuity Loss and Treatment Outcomes

In general, children with JIA-associated chronic anterior uveitis are at high risk for developing ocular complications and visual acuity loss over time. Rates of structural ocular complications (regardless of gender) have ranged from 0.05 to 0.17/PY and rates of loss of visual acuity to the 20/50 or worse and the 20/200 or worse thresholds are estimated as 0.18/eye-year (EY) or 0.20/person-year (PY) and 0.09/EY or 0.14/PY, respectively [19, 24]. Most of the published data reporting long-term clinical outcomes in JIA-associated uveitis focus primarily on patients with chronic anterior uveitis rather than patients with HLA-B27 positive, enthesitis-related JIA. In these cohorts, the majority of patients are girls with frequencies ranging from 70 to 85% on average [19, 21, 24–26]. Despite the lower numbers of boys diagnosed with JIA-related chronic anterior uveitis, several reports have demonstrated worse clinical outcomes among boys [21, 25, 26]. Sabri and colleagues [26] found earlier posterior synechiae formation (*P* = 0.03) and earlier development of any uveitis-related ocular complication (*P* = 0.008) in boys as compared to girls in their single-center, retrospective study of 142 patients (29 were boys) with JIA-associated uveitis. Despite the increased risks of ocular complication, there were no gender-associated differences in visual acuity outcomes during the follow-up period in their study [26]. Ayuso and colleagues [25] and Hoeve and colleagues [27] both reported worse clinical outcomes in boys with JIA uveitis in The Netherlands. In the study by Ayuso et al., 65 children with JIA-associated uveitis, 18 of whom were boys, were analyzed for the development of ocular complications and loss of visual acuity [25]. In the multivariate analysis, boys appeared to be independently associated with an increased risk of cataract surgery (adjusted hazard ratio [HR] = 4.33; *P* < 0.01), cystoid macular edema (adjusted HR = 4.59; *P* = 0.01), and papillitis (adjusted HR = 4.10; *P* = 0.01). These findings were independent of the presence of the HLA-B27 haplotype at the initial evaluation. In another paper from the Netherlands [27], the authors investigated the level of cellular inflammatory activity and the use of anti-inflammatory and immunosuppressive therapy in 62 children with JIA-associated uveitis, 22 of which were boys. Although the authors observed no gender-related differences in the level of cellular inflammation or use of topical corticosteroids, there was a suggestion of an increased use of immunosuppressive drug therapy in boys, particularly during estimated puberty years (*P* = 0.014); however, after correcting for multiple testing using the Bonferroni technique, the findings failed to achieve conventional statistical significance (*P* = 0.112). These findings may suggest a lower threshold for the administration of systemic immunosuppression in boys with JIA uveitis. In a study from the United Kingdom, male sex was found to be independently associated with a higher rate of ocular complications (RR = 4.3; 95% CI: 1.1–16.6; *P* = 0.032), and the rate in boys appeared to be higher in the first four years of followup when compared with girls [21]. After 8 years of followup, approximately 40% of the boys had been diagnosed with at least one ocular complication versus 10% of the girls. Boys had an increased risk of ocular complications in the standard cohort (*P* = 0.001), which was the group of patients that were diagnosed with uveitis by the authors as a result of their screening program (e.g., incident cases of JIA uveitis). Interestingly, there was no association between the development of ocular complications and gender in the nonstandard cohort in which the patients were diagnosed prior to referral to the authors. Thus the nonstandard cohort was subject to referral bias and included patients referred with ocular complications and those whose uveitis presented prior to or at the same time as the joint disease [12].

Two studies that reported on the incidence of visual loss and ocular complications in JIA uveitis also have suggested that boys are more likely to have uveitis diagnosed before or simultaneously to the development of joint disease [25, 27]. Time between the diagnosis of arthritis and uveitis was reported as shorter in boys (0.3 years) than in girls (1.0 years; *P* = 0.02) [25]. Additionally, boys presented with uveitis as the initial manifestation of JIA more frequently than girls (44% of boys versus 15% of girls; *P* = 0.02) [25].

Although studies that specifically look at the differences between clinical outcomes according to gender have been limited, it is possible to make some inferences from additional retrospective cohort studies [19, 20, 24]. For example, Gregory and colleagues reported the clinical outcomes of patients with JIA-associated chronic uveitis from SITE Cohort Study [19]. In this study of 327 patients, active uveitis statistically significantly increased the risk for developing visual acuity loss while use of methotrexate was associated with a reduced risk of developing such visual loss. Both of these findings were independent of gender suggesting that boys and girls had similar risks for developing loss of visual acuity and responded to treatment with methotrexate similarly [19]. Similar findings regarding active uveitis and the effect of methotrexate were reported by Thorne and colleagues in 75 patients with JIA-associated uveitis [24]. Furthermore, we were able to reanalyze the data from the Johns Hopkins cohort with a specific interest in difference in ocular complications and treatment outcomes according to gender. The incidence rates of ocular complications during followup (including ocular hypertension, posterior synechiae, band keratopathy, hypotony, cataract, macular edema, and optic nerve edema) and of visual acuity loss were not statistically significantly different between girls and boys. Again, the studies in which registry data or newly diagnosed JIA uveitis cases were analyzed seemed to suggest higher complication rates in boys compared to girls; however, the type of outcomes differed from study to study making comparisons between studies difficult. Larger prospective studies that would allow for direct comparison of newly diagnosed patients with JIA uveitis and using standardized outcomes and treatment guidelines would provide helpful data to better ascertain any gender-related differences in disease severity and response to therapy.

## 5. Conclusions

Although the typical chronic anterior uveitis associated with JIA occurs predominantly in girls, several reports have demonstrated worse clinical outcomes among boys; however, these findings are not consistent across all studies and limited data are available from prospective or population-based studies. Further studies, particularly studies with prospectively acquired standardized data and outcome measures, are needed to further clarify the role of gender in JIA-associated uveitis and its clinical course.

## Figures and Tables

**Table 1:** Summary of some of the cohort and population based studies for the prevalence and/or incidence of juvenile idiopathic arthritis-associated uveitis.

Study	Design	Population at risk	Prevalence			Incidence			Country	Beginning	Study period
			N of patients, (rate/100,000)			N of patients, (IR*)		
			Total	F	M	Total	F	M
Nordic Countries Cohort [4]	Prospective population-based	1,407,315	N/A	N/A	N/A	315 (15)	197	118	Nordic countries	July 1, 1997	1.5 years
Alsace, France Cohort [5]	Retrospective epidemiologic study	339,095	(19.8)	N/A	N/A	67 (3.2)	N/A	N/A	Northeastern France	January 1, 2001	1 year
Estonia Cohort [6]	Population-based study	262,284	N/A	N/A	N/A	162 (21.7)	86 (22.9)	76 (19.3)	Northernmost of the Baltic States	January 1, 1998	3 years
CHU de Poitiers Cohort [7]	Survey among pediatricians	305,198	48 (15.7)	29 (N/A)	17 (N/A)	N/A	N/A	N/A	Western France	2006	
Prague Cohort [8]	Population-based study	31,703	43 (140)	28 (29.3)	15 (16.6)	4 (13)	2 (2.1)	2 (2.2)	Czech Republic	March 1, 2012	1 year
Catalonia Cohort [9]	Prospective, population-based study	2,119,382	432 (39.7)	288 (N/A)	144 (N/A)	145 (6.9)	93 (9.0)	52 (4.8)	Northeast Spain	2004	2 years
Norwegian Cohort [10]	Population-based multicenter study	255,303	N/A	N/A	N/A	36 (14)	21 (17)	15 (11)	Southeastern Norway	June 1, 2004	1 year

F: female; IR: incidence rate; M: male; N: number.

*Per 100,000 person/year.

**Table 2:** Summary of ocular complications during followup in some of the noted studies.

	Heiligenhaus et al. 2005 [11]	Edelsten et al. 2003 [12]	Saurenmann et al. 2007 [14]	Gregory et al. 2013 [19]	Woreta et al. 2007 [20]	Thorne et al. 2007 [24]	Edelsten et al. 2002 [21]	Ayuso et al. 2010 [25]	Sabri et al. 2008 [26]
Mean ± SD/median age at the time of diagnosis of uveitis	5.2 ± 3.2 Y	N/R	8.3 Y	4.1 (4 m–16.9) Y	7 (1–36) Y	7 (1–36) Y	50 (4–149) m	M: 4.3 (1.7–13.8) Y F: 4.2 (1.7–16) Y	N/R
Follow-up time for uveitis, Mean ± SD/median (range)	5.6 ± 4.9 Y	N/R	6.3 (0–23.2) Y	2.62 (0–24) Y		3 (1 m–15) Y	4 Y	M: 11(1.1–27.5) Y F: 7.2(1.1–22.9) Y	6.3 (0.1–23.2) Y
Type of uveitis									
Anterior	N/R	70%	96.5%	N/R	86.7%	86.7%	N/R	99%	96.5%
Acute anterior	N/R	16%	16.2%	N/R	N/R	N/R	N/R	N/R	16.2%
Chronic anterior	N/R	54%	68.3%	N/R	N/R	N/R	N/R	N/R	68.3%
Anterior recurrent uveitis	N/R	0	12%	N/R	N/R	N/R	N/R	N/R	12%
Intermediate	N/R	0	0	N/R	13.3%	13.3	N/R	0	0
Posterior	N/R	30%	0	N/R	0	0	N/R	0	0
Panuveitis	N/R	N/R	3.5%	N/R	0	0	N/R	1%	3.5%
Structural ocular complications									
At least one OC at presentation	45%	N/R	N/R	60.2%	67%	67%	N/R	N/R	N/R
At least one OC after F/U	56%	N/R	37.3%	N/R	N/R	0.33 EY	21%	N/R	32.5%
Band keratopathy	29%	N/R	14.1%	32.1% (126/393)*	31.5%^¥^	0.08/EY	N/R	16%	12.3%
Posterior synechiae	27%	N/R	21.8%	22.9% (97/423)*	27.5 %^¥^		N/R	40%	17.1%
Cataract	26%	N/R	23.2%	N/R	22.5%^¥^	0.04/EY	21%	42%	20.2%
Glaucoma	8%	N/R	15.5%	N/R		0.05/EY	14%	25%	
Ocular hypertension ≥ 21 mm Hg	N/R	N/R	N/R	24.6 % (125/508)*	15.3%^¥^	0.18/EY	N/R	N/R	13.6%
Ocular hypertension ≥ 30 mm Hg	N/R	N/R	N/R	10.6% (61/577)*			N/R	N/R	
Hypotony	N/R	N/R	N/R	10.5% (60/569)*	9.3%^¥^	0.09/EY	N/R	N/R	N/R
Cystoid macular edema	6%	N/R	4.9%	8.9% (50/564)*	3%^¥^	0.04/EY	N/R	19%	3.9%
Epiretinal membrane	N/R	N/R		8.1% (46/567)*	3.8%^¥^	0.06/EY	N/R	N/R	N/R
Final VA < 20/50 (visual impairment)	31%	N/R	3.4%	36.8% (131/356)*	36.4%	0.10/EY	11%	N/R	3.4%
Final VA < 20/200 (Blindness)	12%	17%	5.7%	21% (95/452)*	23.7%	0.08/EY	6%	N/R	5.7%

EY: eye-year; F: female; F/U: followup; IR: incidence rate; M: male; m: months; N: number; N/R: result not reported; OC: ocular complications; VA: visual acuity; Y: years.

*Incidence; ^¥^prevalence at presentation.

**Table 3:** Summary of gender specific findings in patients with JIA-associated uveitis in some of the recent published articles.

Author/year	Males were more likely to have	Females were more likely to have
Saurenman et al./2007 [14]	(i) Symptomatic uveitis (ii) A shorter interval between diagnosis of arthritis and uveitis (iii) An older age at diagnosis of JIA (iv) Enthesitis-related arthritis and psoriatic JIA.	(i) Female sex was a risk factor for the development of uveitis in patients with oligoarticular and persistent oligoarticular JIA, but not in those with the other subtypes. (ii) Females were more likely to have oligoarticular JIA.

Saurenman et al./2010 [18]		(i) Only girls had an age-dependent and ANA-associated increased risk of uveitis. (ii) Female sex (LR 8.0, *P* = 0.004) and the combination of female sex and young age at diagnosis (LR 6.7, *P* = 0.009) were significantly associated with the development of uveitis. (iii) Girls had a higher rate of ANA positivity compared with boys, in all age groups (*P* < 0.0001).

Sabri et al./2008 [26]	(i) Male patients tended to have a greater rate of uveitis complications (48.3% vs 33.5% in female patients); this difference was not statistically significant (*P* = 0.17). (ii) Male patients were more likely to have earlier complications than female patients (*P* = 0.008), including earlier synechiae formation (*P* = 0.03).	The majority of patients who developed uveitis (79.6%) was female.

Ayuso et al./2010 [25]	(i) Male gender appeared to be independently associated with cataract surgery CME and papillitis (*P* < 0.05). (ii) Time between the diagnosis of arthritis and uveitis was shorter in boys than in girls (0.3 vs 1.0 years). (iii) Boys presented with uveitis as initial manifestation of JIA more frequently than girls (44% vs 15% of girls).	

Hoeve et al./2012 [27]	Uveitis in boys was more frequently diagnosed before the onset of arthritis.	Girls were significantly younger than boys at diagnosis of JIA; however, the age at diagnosis of JIA-associated uveitis was similar for boys and girls.

Edelsten et al./2002 [21]	(i) Male sex was associated with increased complications in the standard cohort. (ii) Male sex was found to be independently associated with a higher complication rate, and the rate appeared to be higher in the first 4 years. (iii) After 8 years the complication rate was 38.4% for males and 9.7% for females.	Females in the standard cohort had the most benign disease with 5% complication rate and 54% remission rate.

Zulian et al./2002 [22]	Males were more susceptible to severe ocular involvement.	

Chia et al./2003 [23]	(i) Male children are more likely to have severe uveitis at diagnosis. (ii) Male patients developed uveitis at shorter intervals from the onset of arthritis symptoms (median 5 vs 18 months).	

CME: cystoid macular edema; JIA: juvenile-associated arthritis; vs: versus.
